# Cardiac optogenetics: using light to monitor cardiac physiology

**DOI:** 10.1007/s00395-017-0645-y

**Published:** 2017-08-31

**Authors:** Charlotte D. Koopman, Wolfram H. Zimmermann, Thomas Knöpfel, Teun P. de Boer

**Affiliations:** 1 Department of Medical Physiology, University Medical Center Utrecht, Yalelaan 50, 3584CM Utrecht, The Netherlands; 2 Hubrecht Institute, Royal Netherlands Academy of Arts and Sciences (KNAW), University Medical Centre Utrecht, 3584CT Utrecht, The Netherlands; 3 Institute of Pharmacology and Toxicology, University Medical Center Göttingen, Göttingen, Germany; 4 DHZK (German Center for Cardiovascular Research), Partner Site, Göttingen, Germany; 5 Laboratory for Neuronal Circuit Dynamics, Imperial College London, London, UK; 6 Centre for Neurotechnology, Institute of Biomedical Engineering, Imperial College London, London, UK

**Keywords:** Physiology, Calcium cycling/excitation–contraction coupling, Ion channels/membrane transport, Cell signalling/signal transduction

## Abstract

Our current understanding of cardiac excitation and its coupling to contraction is largely based on ex vivo studies utilising fluorescent organic dyes to assess cardiac action potentials and signal transduction. Recent advances in optogenetic sensors open exciting new possibilities for cardiac research and allow us to answer research questions that cannot be addressed using the classic organic dyes. Especially thrilling is the possibility to use optogenetic sensors to record parameters of cardiac excitation and contraction in vivo. In addition, optogenetics provide a high spatial resolution, as sensors can be coupled to motifs and targeted to specific cell types and subcellular domains of the heart. In this review, we will give a comprehensive overview of relevant optogenetic sensors, how they can be utilised in cardiac research and how they have been applied in cardiac research up to now.

## Introduction

In recent years, the term optogenetics has become synonymous with research that applies channelrhodopsins to trigger depolarisation of cells by exposure to blue light. This is, however, a rather narrow definition that disregards the broad range of possibilities that arises from combining genetic strategies with optical techniques. In this review, we will conform to the wider definition suggested by Gero Miesenböck that optogenetics “combines genetic engineering with optics to observe and control the function of genetically targeted groups of cells with light” [47]. While already widely used in neuroscience [19, 43], optogenetic methods are now slowly finding their way to cardiac physiology laboratories. In our view, optogenetics has the potential to resolve cardiac physiology in a so far unprecedented way. Cardiac applications of optogenetic actuators such as channelrhodopsin have been covered by several recent reviews [7, 11, 16]. Here, we will review optogenetic sensors that are particularly relevant to the cardiac field, studies that have applied optogenetics in the heart, and outline some of the research questions that can be addressed using optogenetic probes and sensors.

## The unique potential of cardiac optogenetics

Our understanding of cardiac physiology owes much to the development of fluorescent organic dyes that allow the study of intracellular ions (e.g., Ca^2+^, Mg^2+^, Na^+^), transmembrane potential or pH using light microscopy. This optic approach has major advantages over ion-sensitive electrodes that were used before availability of these dyes. It became possible to study many regions of a specimen concurrently without impalement of individual cells and it enabled studies of subcellular mechanisms, including the spatial and temporal resolution of calcium sparks or mitochondrial membrane potential changes, which is impossible with available microelectrodes.

While organic dyes are powerful tools, application of these dyes has practical limits, some of which can be mitigated using an optogenetic approach instead. The main obstacle of using organic dyes is that the experiment can be done only once. After staining a specimen with a dye, the dye will diffuse within the tissue, accumulate in intracellular vesicles or is lost in another way. This results in a decreased specificity of the fluorescent signal, meaning that the staining will typically have to be repeated on a new specimen.

As an additional obstacle, organic dyes can only be used on isolated hearts or isolated cardiomyocytes and are not suitable for in vivo studies. Clearly, in vivo experiments will provide a better understanding of the complex physiology of the heart and the way it functions within the context of the whole body. Also, minimally invasive in vivo experiments may be repeated over time using the same animals, giving the study a greater power to discriminate between experimental groups.

**Fig. 1:**
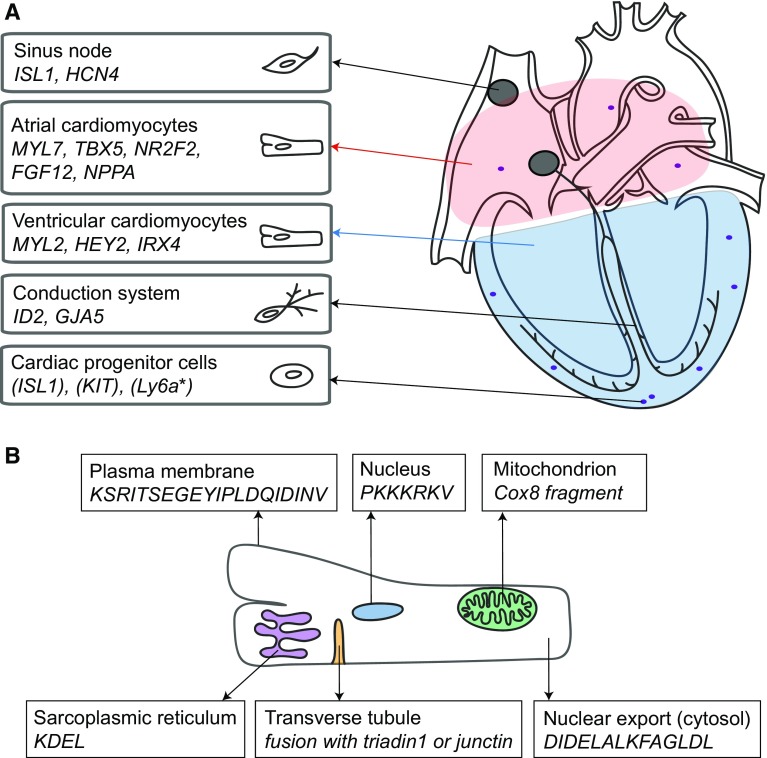
Motifs to target specific cardiac cells or cell organelles. **a** Overview of genes that are higher expressed in subareas of the heart and can be used to target specific cells [73]. The *red region* indicates atrial cells, the *blue region* ventricular cells. Genes from cardiac progenitor cells are between brackets, since it is unclear if they are indeed progenitor cell specific. Gene names can differ between species and gene expression may be dependent on developmental stage and specie. *Ly6a is only found in the mouse. **b** Overview of motifs that can be used to target specific locations within the cell

By employing optogenetic sensors and expressing them in the heart, serial in vivo investigation of cardiac parameters such as intracellular calcium, pH or membrane potential is conceivable. Moreover, by utilising specific targeting motifs, sensors or actuators can be designed to mark subcellular domains and functions in specific cell types in the heart. Protein targeting motifs for the sarcoplasmic reticulum, plasma membrane, cytoplasm, lysosomes, nucleus, transverse tubule and mitochondrion are readily available. An overview of available cell-specific promoters and compartment-specific motifs can be found in Fig. 1.

A clear advantage for the application of optogenetic tools in neuroscience is the lack of gross movements of the brain, opposed to the continuous cardiac cycle that complicates conventional imaging approaches in cardiac physiology. Some possible solutions to this technical challenge will be discussed in later sections of this review.

## The building plan of optogenetic sensors

Optogenetic sensors are generally composed of a sensing domain linked to one or more fluorescent proteins (FPs). The sensing domain can be activated by the parameter of interest, causing a conformational change. This in turn affects the optical properties of the FPs, either through altering the fluorescence quantum yield (brightness) of a single FP or by giving rise to changes in Förster resonance energy transfer (FRET) efficacy between two FPs.

This general building plan of optogenetic sensors offers great flexibility and allows numerous sensor compositions. Sensing domains are often based on fragments of endogenous proteins that interact with or respond to the parameter of interest. By altering the sensing domain, its specificity or binding kinetics can be tuned to match the scientific question. Recent advances have increased the number of FPs and the colours that can be used. This has for instance resulted in multiple calcium sensors with different colours [78] and in FPs that provide sensors with stronger FRET responses [37]. Detailed discussion of the structural composition of optogenetic sensors and actuators is beyond the scope of this review, for such information we refer the reader to recent excellent reviews on that topic [5, 34].

## Optogenetic sensors for cardiac research

**Fig. 2:**
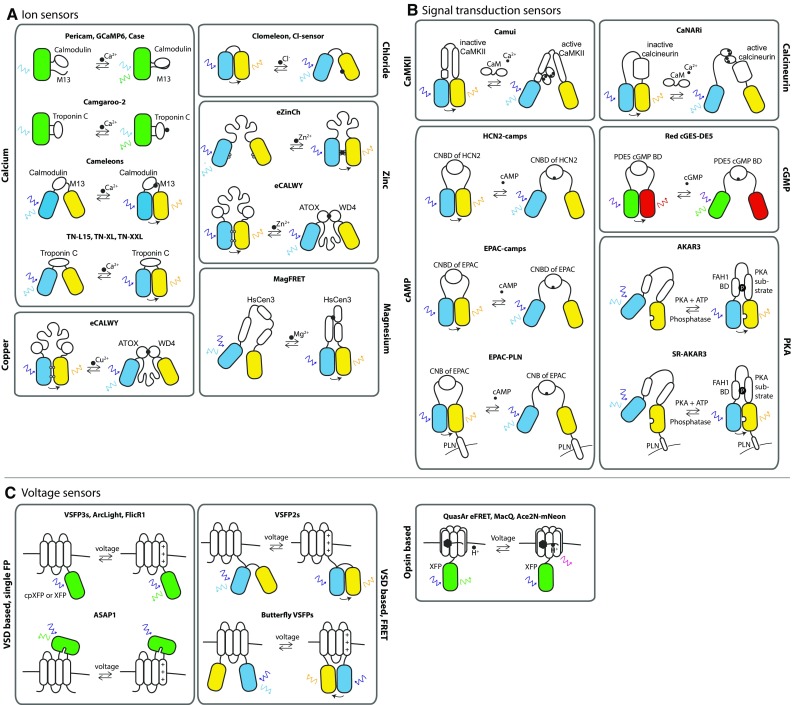
Overview of cardiac optogenetic sensor designs. **a**–**c** each give an overview of a group of sensors that are relevant for cardiac research. For each sensor its mode of action is schematically visualised. Fluorescent proteins are depicted as *coloured barrels*, proteins or protein domains as *white barrels*. *Coloured arrows* indicate excitation/emission wavelengths. **a** Overview of optogenetic ion sensors. These sensors are based on proteins that can sense and bind the ion of interest with a high affinity. Upon binding, a conformational change occurs within the sensor, inducing or diminishing fluorescence or FRET [14, 27–29, 35, 36, 40, 43, 44, 49, 50, 60, 72]. **b** Overview of optogenetic signal transduction sensors. These sensors consist of proteins or substrates that can bind the signalling molecule of interest. In case of a substrate, binding will result in activation or deactivation of the substrate. Ultimately, a conformational change of the sensor will induce or diminish fluorescence or FRET [6, 41, 45, 52, 53, 61, 63]. **c** Overview of optogenetic voltage sensors. VSD-based sensors are composed of a voltage-sensing transmembrane protein linked to either a single fluorescent protein or to a FRET fluorescent protein pair. When the membrane charges, the VSD displaces, giving rise to a fluorescent response [32, 42, 48, 62]. Opsin-based voltage sensors are based on microbial rhodopsin proton pumps and fluorescence is induced via a voltage-dependent shift in the acid–base equilibrium of the retinal Schiff base located in the proton pump [24, 25, 79]

An overview of the sensors that will be discussed in this section is given in Fig. 2 and Table 1.

**Table 1:** List of the latest GEVIs apt for cardiac in vivo studies

Name sensor	Year of publication	Design type	Monochromatic or FRET based	Source of voltage-sensing domain or opsin	Expression system for functional characterization	Readout chromophore’s peak emission wavelengths	Sensitivity (%Δ*R*/*R* per 100 mV) (%)	Response time constant *τ* (on)	Response time constant *τ* (off)	Reference
ArcLight A242, Q239	2012	VSD class	Monochromatic	Ci-VSP	HEK293 cells and cultured neurons, fruitfly in vivo	Super ecliptic pHluorin A227D	35	14.5 ms (122 ms)	44.6 ms (273 ms)	Jin et al. [32]
VSFP2.3	2008, 2015	VSD class	FRET	Ci-VSP	Mouse heart in vivo and ex vivo, PC12 cells	mCerulean: 477 nm mCitrine: 530 nm	15	3 ms (16 ms)	31 ms	Lundby et al. [42], Liao et al. [13]
Mermaid	2008, 2010	VSD class	FRET	Ci-VSP	Mouse heart ex vivo, zebrafish heart	mUKG: 499 nm mKOκ: 563 nm	±30	5–20 ms	5–20 ms	Tsutsui et al. [66, 67], Kaestner et al. [33]
Chimeric VSFP-butterfly	2014	VSD class	FRET	Ci-VSP/KV3.1 chimera	HEK293 cells, mouse in vivo	mCerulean: 477 nm mCitrine: 529 nm mCitrine: 529 nm mKate2: 633 nm	14.7 12.7	2.1 ms (36.7 ms) 2.3 ms (81.2 ms)	14.6 ms 25.1 ms	Mishina et al. [48]
VSFP-CR	2012, 2017	VSD class	FRET	Ci-VSP	Hippocampal neurons, hiPS-CM	Clover: 515 nm mRuby2: 600 nm	12.7	5.4 ms (59.5 ms)	N.D.	Lam et al. [37], Chen et al. [15]
ASAP-1	2014	VSD class	Monochromatic	Chicken VSP	HEK293 cells and cultured neurons	GFP: 505 nm	±20	2.1 ms (72 ms)	50.8 ms (2 ms)	St-Pierre et al. [62]
QuasAr eFRET	2014	Microbial opsins	Monochromatic	Archaerhodopsin	HEK293 cells and cultured neurons	EGFP: 505 nm Citrine: 530 nm mOrange: 562 nm mRuby2: 600 nm mKate2: 633 nm	−7.7 −13.1 −10 −8.7 −4.5	4.3 ms (27 ms) 4.8 ms (21 ms) 4.3 ms (26 ms) 4.3 ms (27 ms) 2.8 ms (35 ms)	3.0 ms (26 ms) 3.1 ms (21 ms) 3.9 ms (27 ms) 3.6 ms (20 ms) 4.0 ms (25 ms)	Zou et al. [79]
MacQ	2014	Microbial opsins	Monochromatic	*L. maculans* rhodopsin	HEK293T cells, cultured neurons, mouse brain slices, mouse in vivo	mCitrine: 530 nm mOrange2: 562 nm	±20 ±20	2.8 ms (71 ms) 2.9 ms (115 ms)	5.4 ms (67 ms) 3.4 ms (20 ms)	Gong et al. [25]
Ace2 N-mNeon	2015	Microbial opsins	Monochromatic	*Acetabularia acetabulum* rhodopsin	HEK293T cells and cultured neurons, fruitfly in vivo, mouse in vivo	mNeon/	12	0.36 ms (4.2 ms)	0.42 ms (5.2 ms)	Gong et al. [24]
FlicR1	2016	VSD class	Monochromatic	Ci-VSP	HeLa cells, HEK293 cells and cultured neurons	cpmApple/597 nm	±3	3.0 ms (41 ms)	2.8 ms (18 ms)	Abdelfattah et al. [1]

### Optogenetic sensors that detect ions

#### Calcium sensors

Calcium signalling is imperative for cardiomyocyte function and the optogenetic detection of calcium can provide valuable information in cardiac studies. Genetically encoded calcium sensors (GECIs) have improved dramatically in the last 5 years, to the point where they are approaching or exceeding traditional organic dyes in terms of signal-to-noise ratios [75]. It is therefore not surprising that calcium sensors have already made their way into cardiac research. However, some organic dyes still have faster Ca^2+^ binding and unbinding kinetics, something that has to be taken into account when designing experiments. Existing GEVIs include the non-ratiometric CaMP sensors (e.g., GCaMP and RCaMP) and the ratiometric sensors such as cameleon, Twitch and TN-XL.

Excitation–contraction coupling in cardiomyocytes, and thus calcium signalling, is an essential process in cardiac contractility and is strongly influenced by the sympathetic nervous system. However, this interaction is difficult to approach experimentally as it ideally requires an in vivo approach. Tallini et al. [65] demonstrated that expression of the optogenetic calcium sensor GCaMP2 in the mouse heart allows the in situ recording of cardiomyocyte calcium transients. In subsequent work of the same group GCaMP2 has been targeted to endothelial cells to study the in vivo relation between acetylcholine-induced calcium waves in endothelial cells and the subsequent dilation of arterioles [64]. Expression of GCaMP2 was also evident in the cardiac Purkinje fibres, enabling recordings of calcium transients selectively in this cell type. A caveat to the use of GCaMP is the apparent induction of hypertrophy as a result of calmodulin motif overexpression [65], but this can be avoided by an inducible expression system.

Another challenge in cardiac research is the analysis of stem cell functionality after transplantation into the heart. Recently, Shiba et al. expressed GCaMP3 in human embryonic stem cell-derived cardiomyocytes (SDCs) to evaluate their survival after transplantation into guinea pig hearts [58]. Calcium signals that were detected, demonstrated that the SDCs survived transplantation and displayed calcium transients that were synchronised with the surrounding native myocardium.

Detection of calcium signals at specific subcellular domains in cardiomyocytes such as the dyadic space between T-tubules and the sarcoplasmic reticulum is challenging. Calcium sparks occurring at the dyad are fundamental to excitation–contraction coupling, while whole cell calcium transients are the summation of many nearly simultaneous calcium sparks. The calcium spark has been studied intensively, but mainly as spontaneous events happening in resting cardiomyocytes. This limitation is the result of the inability to resolve calcium sparks (small amplitude), or the dyadic space from the rest of the cytoplasm during whole cell calcium transients (large amplitude). However, in a recent publication, Shang et al. demonstrate that dyadic calcium imaging is feasible in rat cardiomyocytes by targeting a GCaMP6 variant to the dyadic space [57]. This approach resulted in an approximately 50× better spatial specificity compared to organic dyes, high contrast and importantly, the ability to study dyadic calcium signalling during the whole excitation–contraction cycle. Another example of targeted calcium sensor was the use of D1ER, a SR targeted version of Cameleon, in neonatal rat cardiomyocytes. Using this approach, the authors demonstrated the role of AKAP18∂ in the regulation of PKA-mediated phosphorylation of phospholamban.

With the possibility to target calcium sensors to specific domains, availability of sensors with different emission wavelengths becomes important. For example dyadic calcium imaging using a targeted green GCaMP6 sensor could be combined with expression of a red calcium sensor targeted to the sarcoplasmic reticulum or cytoplasm to reveal interaction between compartments. Recently, GECIs with blue, orange or red emission have become available [3, 31, 55, 78]. Particularly, the red sensors have attracted attention, as they have the advantage that they can be combined with channelrhodopsins, allowing light-induced pacing with blue light and simultaneous study of calcium signalling with green excitation light and red emission [3]. No studies have been published yet that employ red calcium sensors in the heart, but ongoing work in our laboratory has shown that cardiac calcium transients can be well resolved in transfected neonatal rat cardiomyocytes with these sensors (RCaMP1h, R-GECO1, R-CaMP1.07 and R-CaMP2).

The strength of calcium sensors that are derived from GCaMP is the relatively high signal-to-noise ratio. However, these sensors are not ratiometric since they contain only one fluorescent protein, in contrast to FRET sensors (e.g., cameleon, Twitch or TN-XL). Especially in the heart, it is important to take this into consideration when selecting a sensor, as ratiometric approaches provide a way to deal with cardiac contraction artefacts that otherwise confound results. Unfortunately, responses of FRET sensor are much smaller in amplitude than those of GCaMP type sensors (typically max. 15%), which make it more challenging to resolve the optical Ca^2+^ signals. Currently, an interesting hybrid is being developed by fusion of GCaMP3 with the calcium-insensitive FP mCherry [59]. The resulting sensor (GCaMP-GR) promises an optimal combination of a high signal-to-noise single emission sensor with the possibility to correct for movement-related florescence signals. However, application in the heart has not been demonstrated yet. Recently developed single emission sensors based on CFP variants and reporting PKA activity, membrane voltage or calcium [10, 56] may also be combined with, e.g., yellow or green FPs to yield a dual emission sensor with high signal-to-noise ratio.

In conclusion, the toolkit available for studying cardiac calcium handling has greatly advanced, enabling in situ studies of important physiologic calcium processes (i.e., sympathetic influence on the heart), experimental processes (i.e., stem cell transplant functionality) and of calcium handling within cellular compartments. In addition, it is possible to measure different compartments simultaneously by employing differently coloured sensors.

#### Sensors to detect other ions

Compared to the recent rapid development of GEVIs with improved performance, there are fewer well performing genetically encoded indicators for optogenetic detection of other ions. To our knowledge, there is no sensor available for sodium ions, even though such a sensor would be very interesting given the direct interaction between sodium and other ions (including calcium) via the various sodium co-transporters (Na^+^/Ca^2+^, Na^+^/H^+^, Na^+^/HCO_3_^−^) and the often increased sodium ion concentration in remodelling cardiomyocytes [9, 69, 70].

Chloride ions can be detected using various optogenetic sensors, which are used in neuroscience given the important role of chloride ions in neuronal excitability [20]. In the heart chloride ions may also play a role in cardiac osmotic balance, excitability and remodelling [21], but their role for now remains elusive.

Magnesium ions influence heart rhythm via potassium and calcium ion channels, and are also relevant in cardiac disease and treatment [17]. Using MagFRET to study intracellular Mg^2+^ in the heart may enhance our insight in the ion’s role in normal physiology and cardiac disease.

Other ions that can be detected using optogenetic sensors include Zn^2+^ and Cu^2+^, which is interesting since both ions are implicated in cardiac disease [4]. However, these sensors have not been used to study cardiomyocytes yet.

### Optogenetic sensors to detect signal transduction

Remodelling of the heart during disease is associated with altered activation of several signal transduction pathways, e.g., CaMKII, calcineurin, cAMP/PKA and cGMP/PKG. The essence of many signal transduction pathways is that they transduce extracellular signals into an intracellular signal, allowing cardiomyocytes to respond their environment. Importantly, extracellular signals typically reach cardiomyocytes via the circulation, from which the heart is disconnected in most conventional experimental settings, meaning that signal transduction pathways are deprived of their physiological input. Application of optogenetic sensors to study signal transduction in vivo could potentially help overcome this limitation.

#### Calcium-sensitive pathways

Binding of calcium to calmodulin leads to activation of CaMKII by calmodulin. The state of CaMKII is, therefore, strongly influenced by changes in calcium signalling, such as induced by variations in heart rate or neurohumoral factors [22, 77]. Interestingly, CaMKII activity can be affected by phosphorylation or oxidation. By employing the CaMKII activity sensor Camui and two CaMKII variants that are resistant to phosphorylation or oxidation, it was demonstrated that activation of CaMKII by angiotensin-II and endothelin-I largely depends on oxidation, while isoproterenol and phenylephrine affect CaMKII mainly through phosphorylation [22]. In vivo exploration of CaMKII regulation by application of Camui may be instrumental in improving our understanding of its role in cardiac remodelling and arrhythmogenesis.

After binding calcium, calmodulin can also activate calcineurin, which in turn phosphorylates NFAT and causes it to migrate into the nucleus where it functions as a transcription factor. Calcineurin is implicated in cardiac hypertrophy and failure [77]. A FRET sensor to detect calcineurin activity has been developed and employs a fragment of NFAT [51]. Applications have not extended to cardiac cells yet, but experiments in MIN6 β-cells have revealed strong differences between calcium dependence of cytoplasmic and ER calcineurin signalling [45], raising the question how subcellular calcineurin activity is regulated in cardiomyocytes.

Given the essential interaction of CaMKII and calcineurin pathways with intracellular calcium, it would be interesting to simultaneous monitor CaMKII or calcineurin with calcium. Since Camui and CaNAR sensors are based on cyan and yellow fluorescent proteins they may be combined with green or red calcium sensors, though combination with green calcium sensors will require the use of spectral deconvolution approaches to better separate yellow and green emission.

#### Studying the downstream effects of cardiac innervation

Sympathetic nerve activity and circulating catecholamines are activators of the cardiac β-adrenergic receptor, causing intracellular production of cyclic AMP and PKA-mediated phosphorylation of proteins that subsequently leads to increased heart rate, stronger contractions and faster relaxation of the heart and shortening of the cardiac action potential. FRET sensors detecting cAMP and cGMP have been used to study cardiac adrenergic receptors and their downstream signalling, and are discussed below.

#### cAMP

Intracellular cAMP/PKA signalling is known to be organised into spatial microdomains [41]. Utilising a transgenic mouse expressing the cAMP sensor HCN2-camps, Nikolaev et al. were able to further specify the contributions of β_1_ and β_2_ adrenoceptors [53]: stimulation of β_1_ adrenoceptors caused a rise in cAMP throughout the cardiomyocyte, while β_2_ adrenoceptor stimulation caused only a very local increase in cAMP. In another study, the lipid raft protein caveolin-3 was demonstrated to be important for function of β_2_ adrenergic receptors, by confining β_2_-AR to the T-tubules it ensures cAMP production upon β_2_-AR stimulation [74].

Faster relaxation of the heart upon sympathetic stimulation is the result of enhanced SERCA activity, which pumps Ca^2+^ from the cytoplasm into the sarcoplasmatic reticulum. By targeting Epac1 to SERCA2a through fusion of the sensor with full length phospholamban (PLN), Sprenger et al. were able to demonstrate that SERCA2a and β_1_-adrenoreceptors communicate via a microdomain that is defined by phosphodiesterase 4 (PDE4) activity [61]. Interestingly, transverse aortic constriction disturbed the communication between the β_1_-adrenoceptor and SERCA2a because PDE4 localisation was disturbed, leading to overflow of cAMP beyond the microdomain.

Linking membrane potential to intracellular signalling, a recent study demonstrated that enhancing late Na^+^ current in atrial cardiomyocytes induces cAMP production by triggering adenylyl cyclase activity through a Ca^2+^-dependent mechanism [23], giving insight in a proarrhythmic mechanism that could not have been revealed without an optogenetic sensor.

#### cGMP

Cardiac remodelling in disease often involves multiple organ systems interacting via circulating hormones, for example the atrial natriuretic factor (ANF) which is released by the atria in response to volume overload [18]. In cardiomyocytes, ANF triggers a cGMP-mediated anti-hypertrophic pathway [54]. cGMP levels can be estimated by measuring cyclic nucleotide gated ion currents, but this approach will only report on subsarcolemmal cGMP levels, and is not feasible in vivo as it requires patch clamp electrophysiology.

In their recent study, Götz et al. employed a transgenic mouse expressing the genetically encoded cGMP sensor red cGES-DE5 to provide a first insight in cGMP signalling in intact adult cardiomyocytes [26]. Basal levels of cGMP are very low in cardiomyocytes (about 10 nM) and can be strongly stimulated by C-type natriuretic peptide and ANF. The resting levels are mostly determined by cGMP generation by NO-sensitive guanylyl cyclases and cGMP degradation by PDE3. After giving the mice a hypertrophic stimulus (mild aortic constriction), PDE5 activity had a greater effect on cGMP levels. Targeting of cGMP sensors to the plasma membrane may give more insight in the importance of particulate versus NO-sensitive GCs.

Concluding, optogenetic cGMP sensors can give novel insights in this important pathophysiological signalling pathway, potentially also in in vivo experiments.

#### PKA activity

Changed cAMP levels influence the activity of PKA, altering ion channel phosphorylation and function, which is an important mechanism by which the sympathetic nerve system increases heart rate when needed. Direct confirmation of this important mechanism was provided in a recent study using the PKA activity sensor AKAR3 [76]. The authors found that interventions that increase cAMP levels in sinoatrial node cardiomyocytes cause increased PKA activity, and that the kinetics and magnitude of the PKA activation underlie increases in beating rate.

Using a variant of AKAR3 fused to phospholamban, SR-AKAR3, Liu et al. investigated β-adrenergic regulation of PKA activity at the SR. Interestingly, the authors found that β-adrenoceptor stimulation with isoproterenol had stronger effects on PKA activity than forskolin or 8-bromo-cAMP, both in neonatal and adult cardiomyocytes [41], suggesting highly localised signalling between the receptor and the SR.

### Optogenetic sensors to detect membrane potential

Like calcium sensors, genetically encoded sensors of membrane potential or GEVIs (genetically encoded voltage indicators) have improved greatly during recent years. The key work in this field was mainly motivated by the use in neuroscience. Application of GEVIs in the cardiac field started soon afterwards but remained limited to a few studies [13, 39]. Existing GEVIs fall into two main classes. One class is based on the bacterial rhodopsin to detect changes in voltage, while the second class relies on a voltage-sensing domain (VSD) derived from voltage-sensing proteins. The fluorescent component of GEVIs often consists of single GFP or the CFP-YFP FRET pair, but other colours have also been reported. An overview of current GEVIs with their main characteristics can be found in Table 1 and in Antic et al. [8].

Clinically used drugs can trigger serious undesirable actions, with one of the most life-threatening responses being cardiac arrhythmias. Many pro-arrhythmic drugs affect the heart directly, but may also influence the heart indirectly through the autonomic nervous system or by activating other physiological mechanisms. Therefore, an in vivo approach is required to gain insight in the full effects that pro-arrhythmic drugs may have on cardiac function. In 2010, Tsutsui et al. reported a novel transgenic zebrafish line with myocardial Mermaid expression, a ratiometric GEVI, in which they were able to measure physiological membrane voltage dynamics in unanesthetized and unrestrained zebrafish embryos. To test the effect of hERG inhibitors on cardiac electrophysiology, embryos were treated with Astemizole. Severe cardiac abnormalities were observed, including a complete absence of ventricular contraction. When performing voltage imaging, it was found that electrical activation first appeared near the atrium–ventricle border and then propagated backward into the atrium, demonstrating in a detailed spatiotemporal manner how cardiac conduction was altered [66]. Models like the Mermaid zebrafish could, thus, provide important new cardiac drug-testing tools.

In addition to their use in drug testing, GEVIs offer the unique possibility to gain more insight into (patho)physiological mechanisms of cardiac conduction, both in the developing and adult heart. Recently, Chang-Liao et al. established a stable-transgenic VSFP2.3 mouse model with cardiac specific expression. In vitro experiments on isolated cardiomyocytes and Langendorff-perfused hearts confirmed that sensor recordings reflected cardiac physiology. To record in vivo action potentials, a minimally invasive fibre optic imaging was developed, in which optical fibres were connected to two high-speed cameras. FRET signals where measured in sedated mice treated without and with blebbistatin, which uncouples excitation and contraction. Interestingly, clear and corresponding signals were identified in both groups indicating that this approach is suitable to study cardiac conduction in the presence of normal contraction and blood perfusion [13].

Ongoing work in our laboratory demonstrates that VSFP2.3 and the newer VSFP-Butterfly CY can also successfully be expressed and analysed in zebrafish hearts (unpublished data).

The Mermaid zebrafish line and the VSFP2.3 mouse line demonstrate an important proof of principle and provide evidence for the high potential of GEVIs in cardiac research. In contrast to GECIs that have been developed to a stage where fundamental improvements in performance are neither likely nor required for most experimental designs, development of GEVIs is still in a very active phase. To resolve action potentials, ideal membrane potential sensors have to generate robust signals on a millisecond timescale and have optimal targeting to the plasma membrane. The latest sensors, with improved signal-to-noise ratio and improved kinetics, include VSFP-Butterfly, ASAP1, VSFP-CR, ArchLight, FlicR1 and the FRET-based Arch, Mac and Ace opsins QuasAr eFRET, MacQ eFRET and Ace2N-mNeon (also see Table 1). All are viable candidates for the use in cardiac experiments.

## In vivo cardiac imaging: challenges and potential solutions

**Fig. 3:**
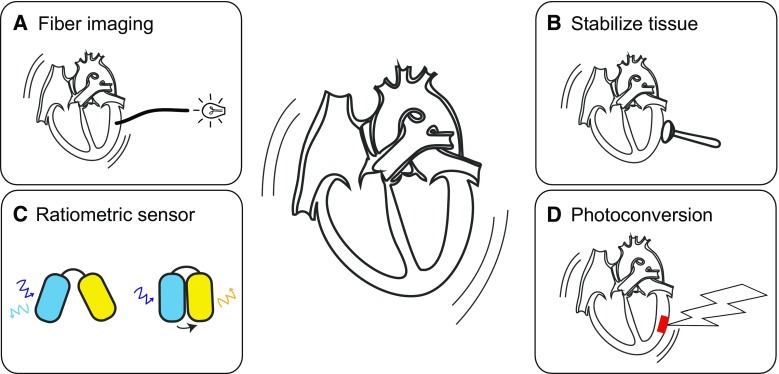
Solutions for cardiac movement artefacts. **a** Optical fibres are flexible and small, thus allowing the local recording of fluorescence even when movement occurs. The fibre ending is directly positioned against the cardiac tissue. **b** When studies are not limited by opening of the thorax, physical immobilisation of cardiac tissue can be achieved by attaching a rigid ring and applying a gentle vacuum. **c** Ratiometric imaging allows compensation of contraction related increases in fluorescence intensity, since it affects the intensities of both the donor and acceptor fluorophore the same. **d** Photoconversion can be used to convert specific cardiac regions and track these during contraction

Application of optogenetic sensors in in vitro experiments has already yielded insights that could not have been obtained otherwise. Yet, moving towards using optogenetic sensors in vivo is even more exciting as it will allow us to study cardiac remodelling in the most realistic setting: as an interaction between heart, brain and kidneys. Ideally, application of optogenetic sensors is combined with optogenetic actuators to, e.g., pace the heart at specific locations, or influence protein–protein interactions [7, 11, 12, 16, 68]. Successful use of optogenetic sensors in vivo requires novel approaches in imaging to deal with the continuous movement and contraction of the heart (see Fig. 3).

### Compensating for myocardial contraction using ratiometric imaging

A tried and tested technique of dealing with cardiac contraction that works well ex vivo is to use ratiometric imaging. In this approach, fluorescent indicators are used that report on the physiological parameter of interest by emitting fluorescence at two wavelengths. Since movement and contractions will affect the fluorescence output equally at the two wavelengths the ratio of the fluorescence intensities at the two wavelengths is therefore, at least theoretically, independent of movement/contraction related indicator signal components. This “rationing” approach is challenging in terms of optical instrumentation since it requires simultaneous imaging at two wavelengths.

Classical examples include FRET dyes like Fura-2 or indo-1, but ratiometric imaging can also be applied to optogenetic sensors, like the FRET sensors Twitch, TN-XL or VSFP2.3. A very interesting approach is to directly fuse a high signal-to-noise optogenetic sensor to a non-sensing fluorescent protein with a different emission wavelength, such as GCaMP-GR [59].

### Optical fibres

During the cardiac cycle, the heart contracts but also rotates, making registration of the site of recording very challenging, even during an ex vivo, Langendorff-perfused experiment. Several labs have used optical fibres to locally record fluorescent emission of dyes or optogenetic sensors. The key benefit of such fibres is their flexibility and small sizes (down to 100 µm or less). Making use of the flexibility of small fibres, our group recently made recordings of calcium transients in vivo, without opening the thorax [46]. By inserting a 250 µm optical fibre through the carotid artery, it was possible to advance the fibre into the left ventricle and record endocardial calcium transients from transgenic mice overexpressing GCaMP3.

### Immobilisation of the heart

Studies that are not limited by opening of the thorax can benefit from the approaches developed to stabilise the heart in situ [2, 38, 71]. The principle of their device is very similar to that of the Octopus heart stabiliser used for human cardiac surgery. Attaching a rigid ring to the heart, either using adhesives or a gentle vacuum, contraction and movement of a small region of the heart is minimised, enabling imaging in living animals. The mechanical stabilisation is combined with electrocardiogram-based triggering, i.e., images are acquired during a selected phase of the cardiac cycle in which the tissue is most stable. While this approach has great potential for in vivo physiology, it is probably not suited for lengthy data acquisition as the tissue may be damaged by prolonged stabilisation.

### Using photoconvertible optogenetic sensors as intrinsic landmarks to track tissue movement

Dealing with the continuous movement of the heart remains a big challenge, despite the available techniques mentioned above. Especially tracking of a region of interest throughout the cardiac cycle is problematic, the vasculature of the heart can be used as a set of landmarks, but still it is difficult to follow regions. To solve this, extrinsic landmarks can be applied, for instance by injecting black ink into the myocardium, or spraying paint on the heart.

The advent of photoconvertible and photoactivatable calcium sensors enables an attractive alternative approach [30]. The photoconvertible sensors GR-GECO1.1 and GR-GECO1.2 can be converted from a green emission calcium sensor into a green and red emission calcium sensor by exposure to ~400 nm light. Similarly, the photoactivatable sPA-GCaMP6 emits very little green light, until it is activated by ~400 nm light, after which it will emit green light to report cytoplasmic calcium concentration. Using such sensors, it would be possible to create intrinsic landmarks within the myocardium, which could be very small, down to the size of a single cardiomyocyte. But also for larger regions that have been converted into a landmark, this approach has several benefits. As the shape of the landmark that has been created is known, tracking movement of that specific region is simplified as one only needs to track the boundaries or pattern of the landmark region. Using this information, it should also be possible to use deformation of the boundaries during contraction to correct for contraction artefacts.

## Conclusion

Application of optogenetic sensors in cardiac research has only just started. The results from the first studies are exciting, and have provided insights that could not have been obtained by conducting experiments using traditional organic dyes. Especially, the ability to target optogenetic sensors to subcellular regions is expected to provide us with many new mechanistic insights in cardiomyocyte physiology.

Small steps have been made towards application of optogenetic sensors in vivo. Further development of this approach will require optimisation of recording strategies. Especially, the continuous movement and contraction of the heart provide a challenge that is not present in other tissues. Anticipated benefits of in vivo optogenetics are the ability to perform longitudinal studies in individual animals, and importantly the possibility to study cardiomyocyte physiology within the context of whole-body physiology.
